# Supplementary material for: Synthesis of Spin-Labeled Ibuprofen and Its Interaction with Lipid Membranes
Source: Molecules. 2022 Jun 27;27(13):4127. doi: 10.3390/molecules27134127 (PMC9268589; doi:10.3390/molecules27134127)
Supplement: Supplementary file 1 [file molecules-27-04127-s001.zip › molecules-1780209-supplementary.pdf]

Supplementary Materials

Synthesis of spin-labeled ibuprofen and its interaction with lipid membrane

Denis S. Baranov,[1*] Anna S. Smorygina,[1] Sergei A. Dzuba[1,2*]

[1]V.V. Voevodsky Institute of Chemical Kinetics and Combustion, SB RAS, 630090 Novosibirsk, Russian Federation

[1,2]Novosibirsk State University, 630090 Novosibirsk, Russian Federation

Table of Contents

NMR spectra……………………………………………………...……………….. S2

IR spectra ……..…………………………………………………………………… S15

HRMS spectra……………………………………………………………………….. S21

NMR spectra

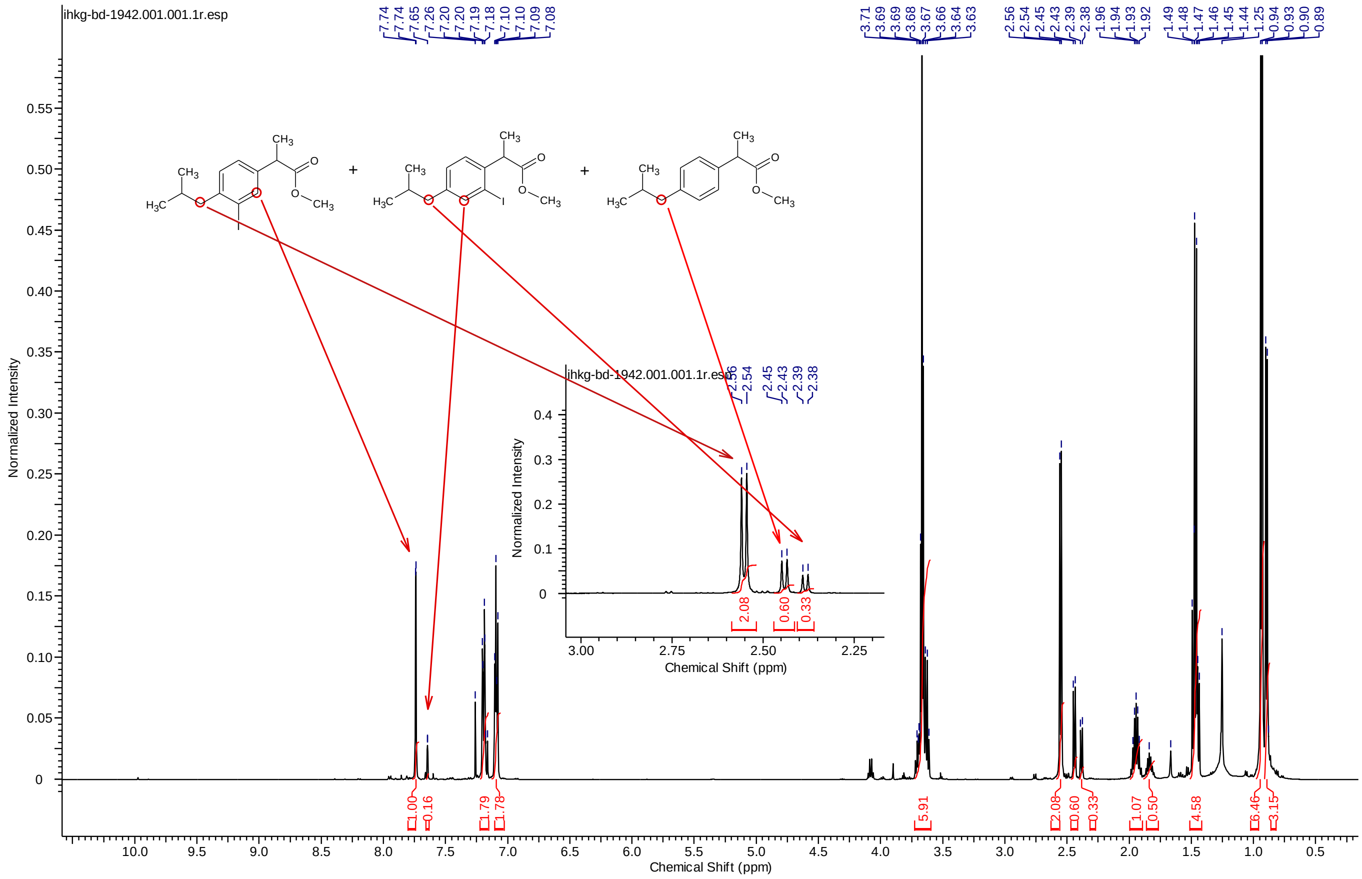

Figure S1. ^{1}H-NMR spectrum (500 MHz, $CDCl_3$) of a mixture of products isolated by the iodination reaction of ibuprofen with iodine and iodic acid in acetic acid at 70 °C, followed by methylation with methanol.

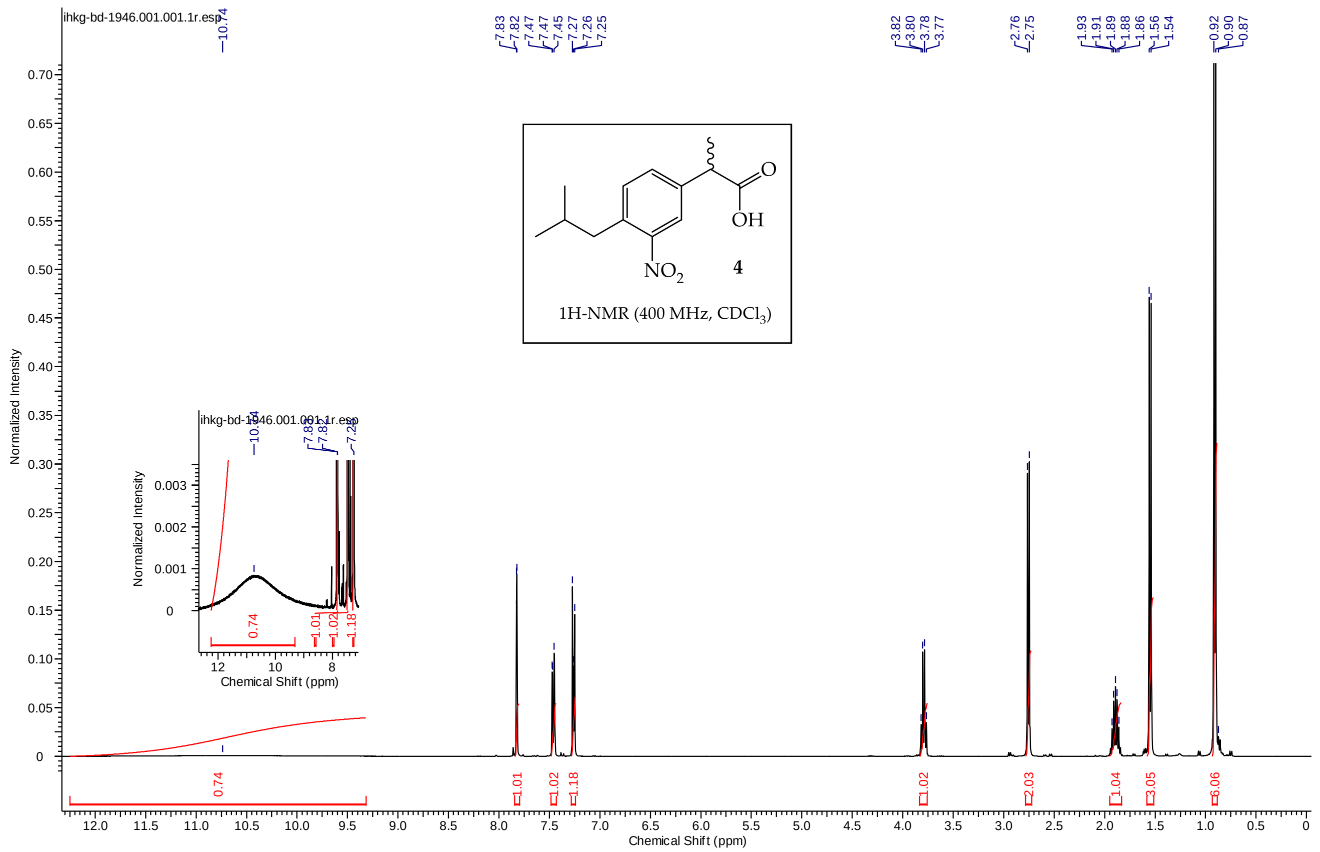

Figure S2. ^{1}H-NMR spectrum of compound **4**.

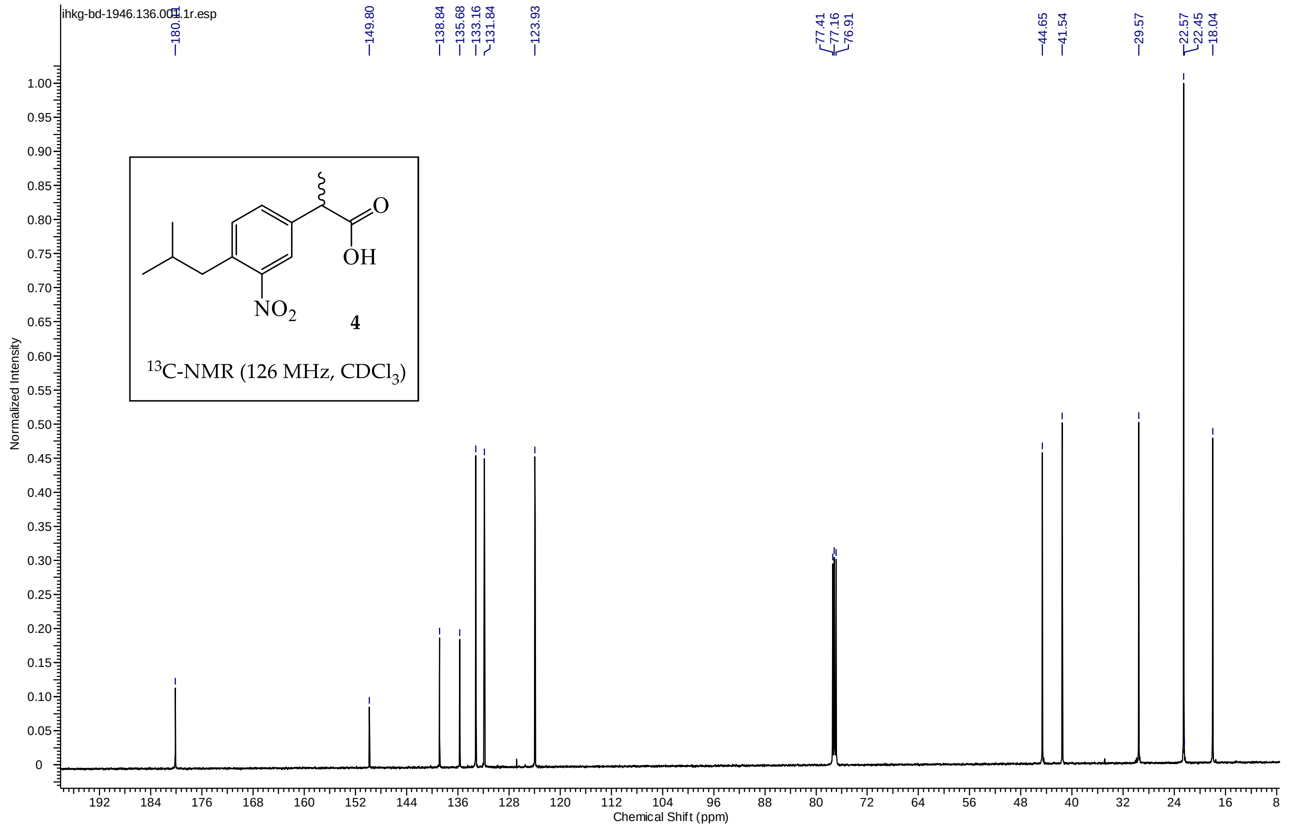

Figure S3. ^{13}C-NMR spectrum of compound **4**.

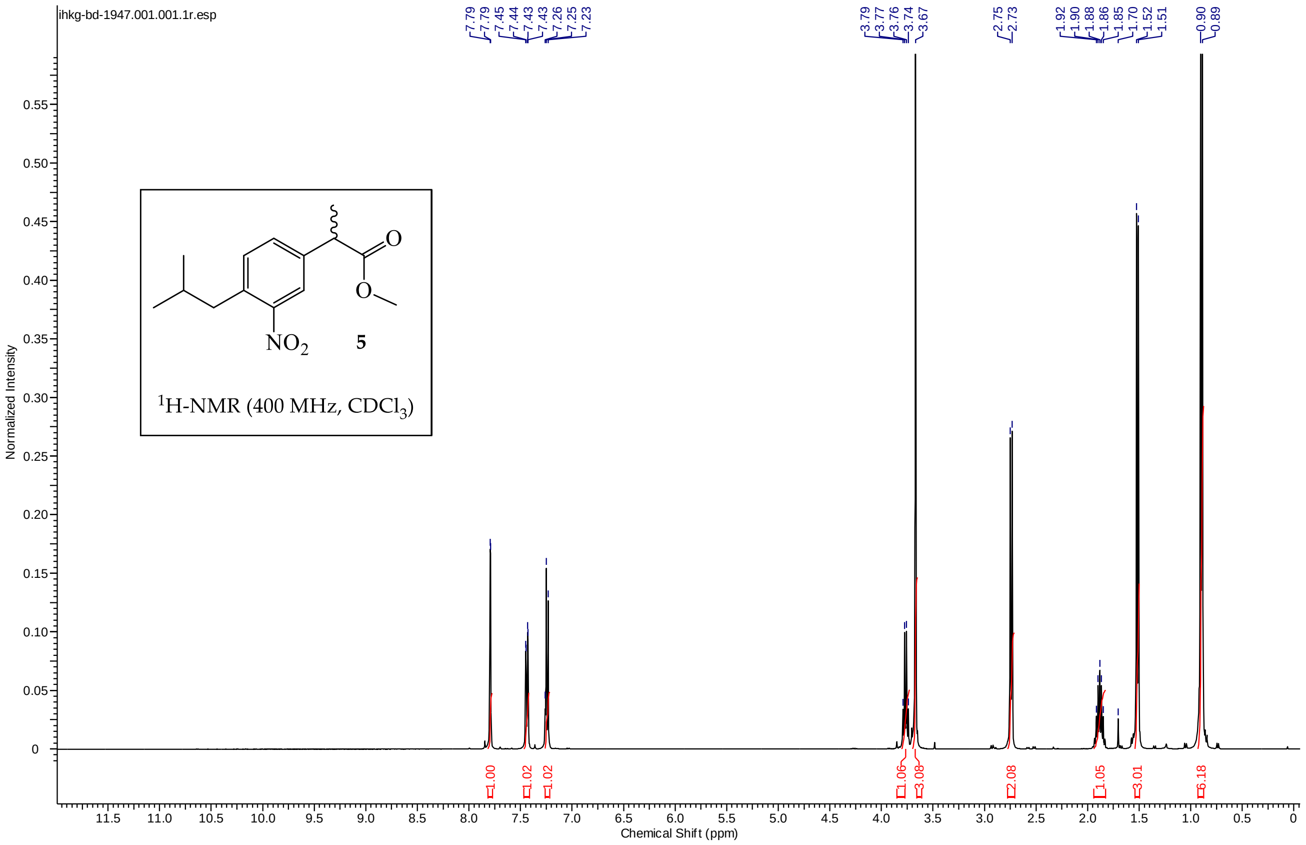

Figure S4. ^{1}H-NMR spectrum of compound **5**.

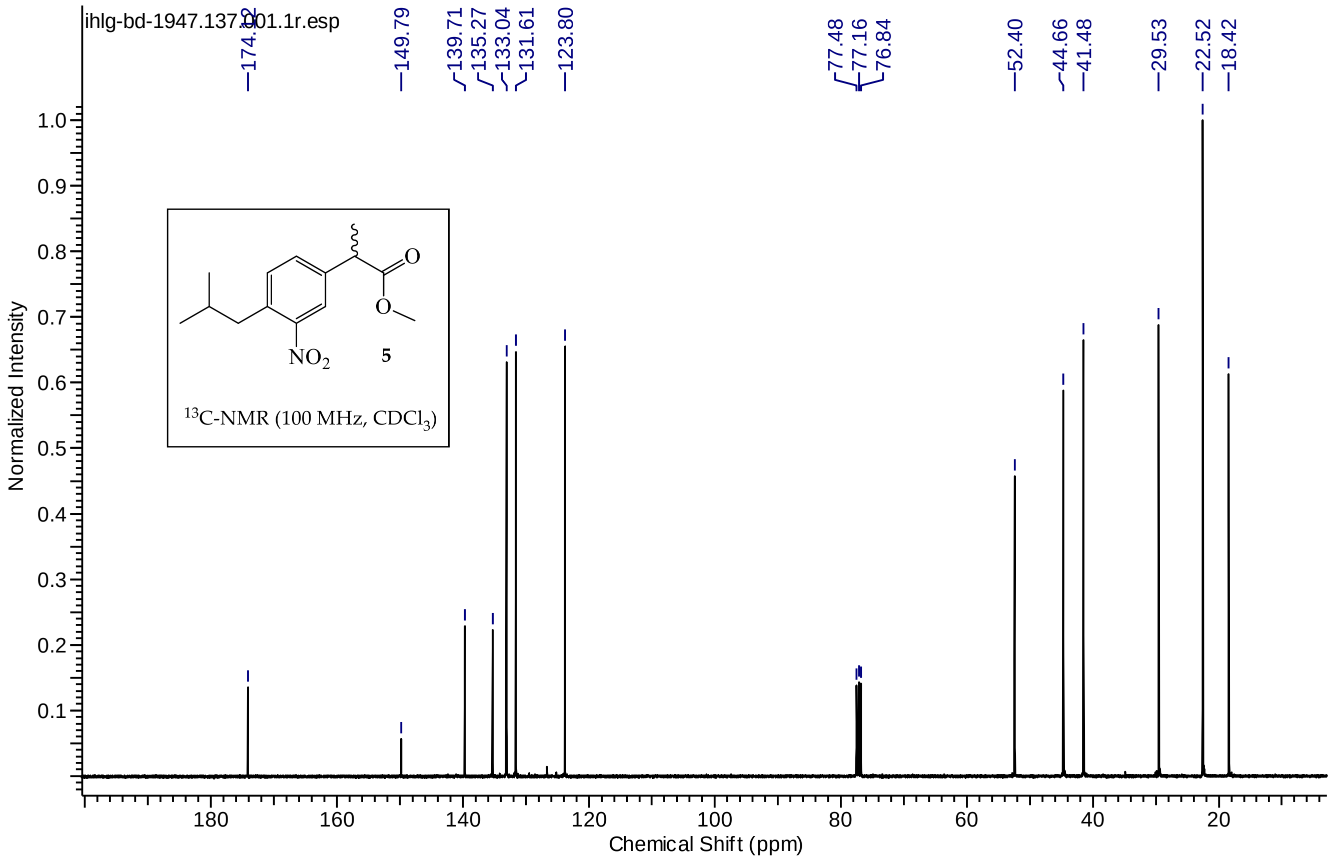

Figure S5. ^{13}C-NMR spectrum of compound **5**.

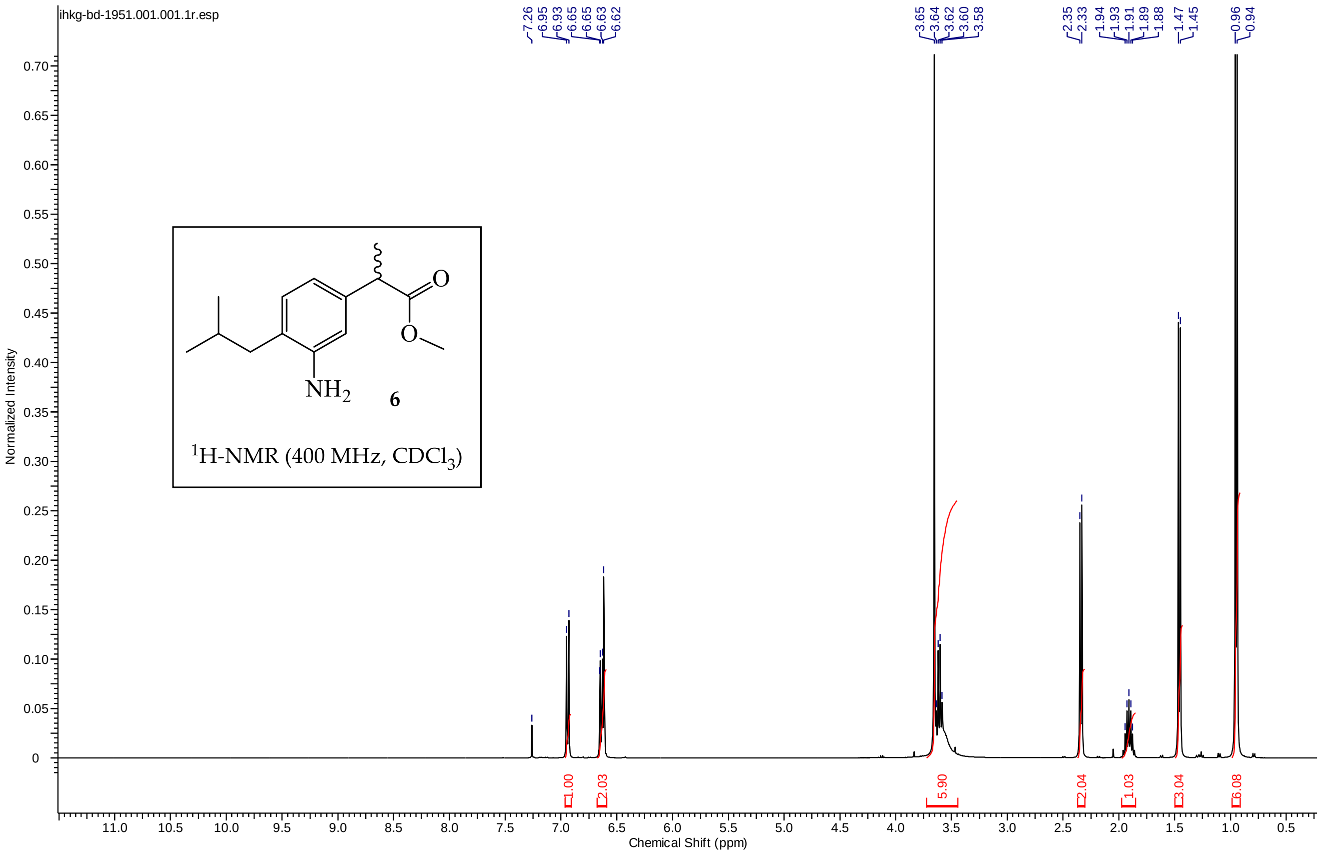

Figure S6. ^{1}H-NMR spectrum of compound **6**.

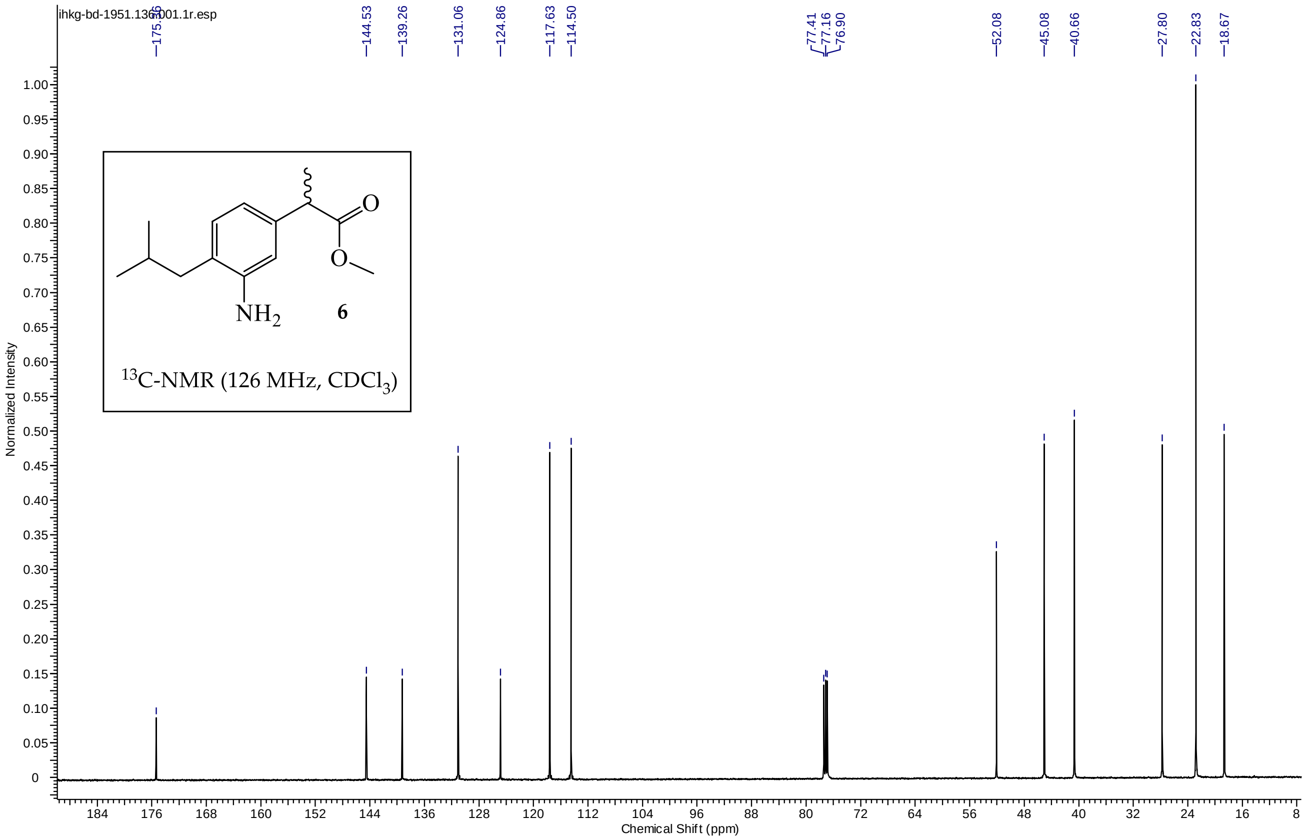

Figure S7. ^{13}C-NMR spectrum of compound **6**.

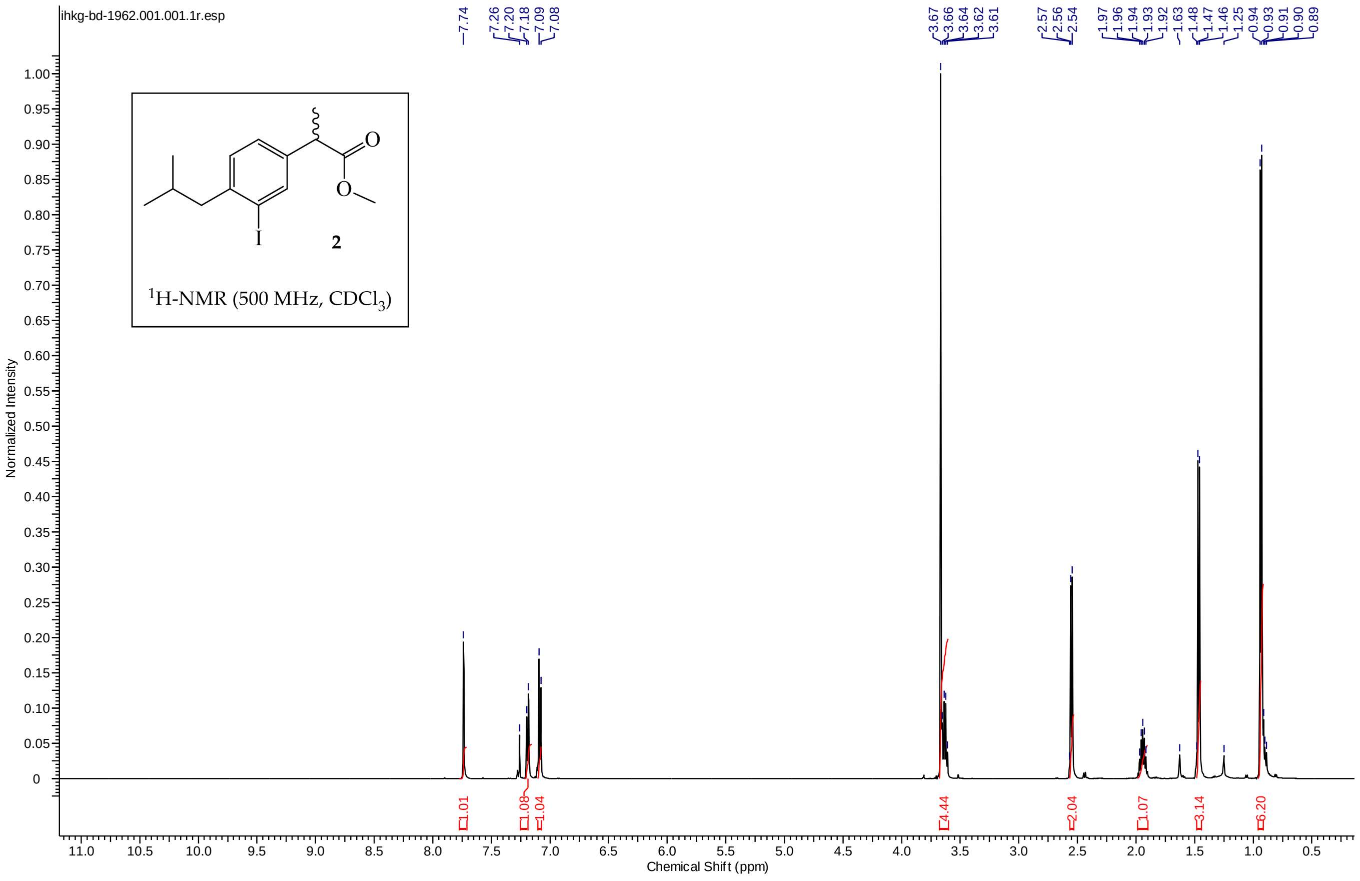

Figure S8. ^{1}H-NMR spectrum of compound **2**.

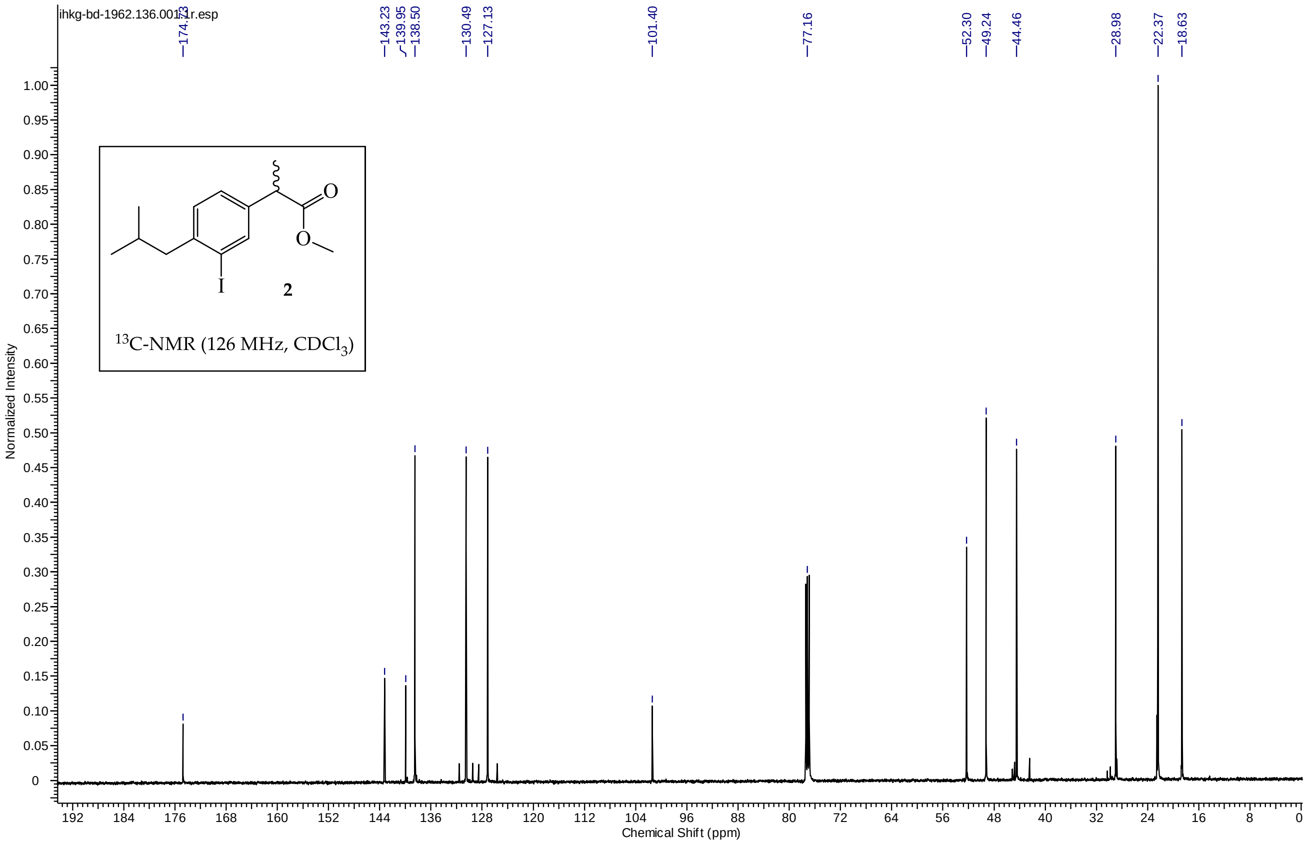

Figure S9. ^{13}C-NMR spectrum of compound **2**.

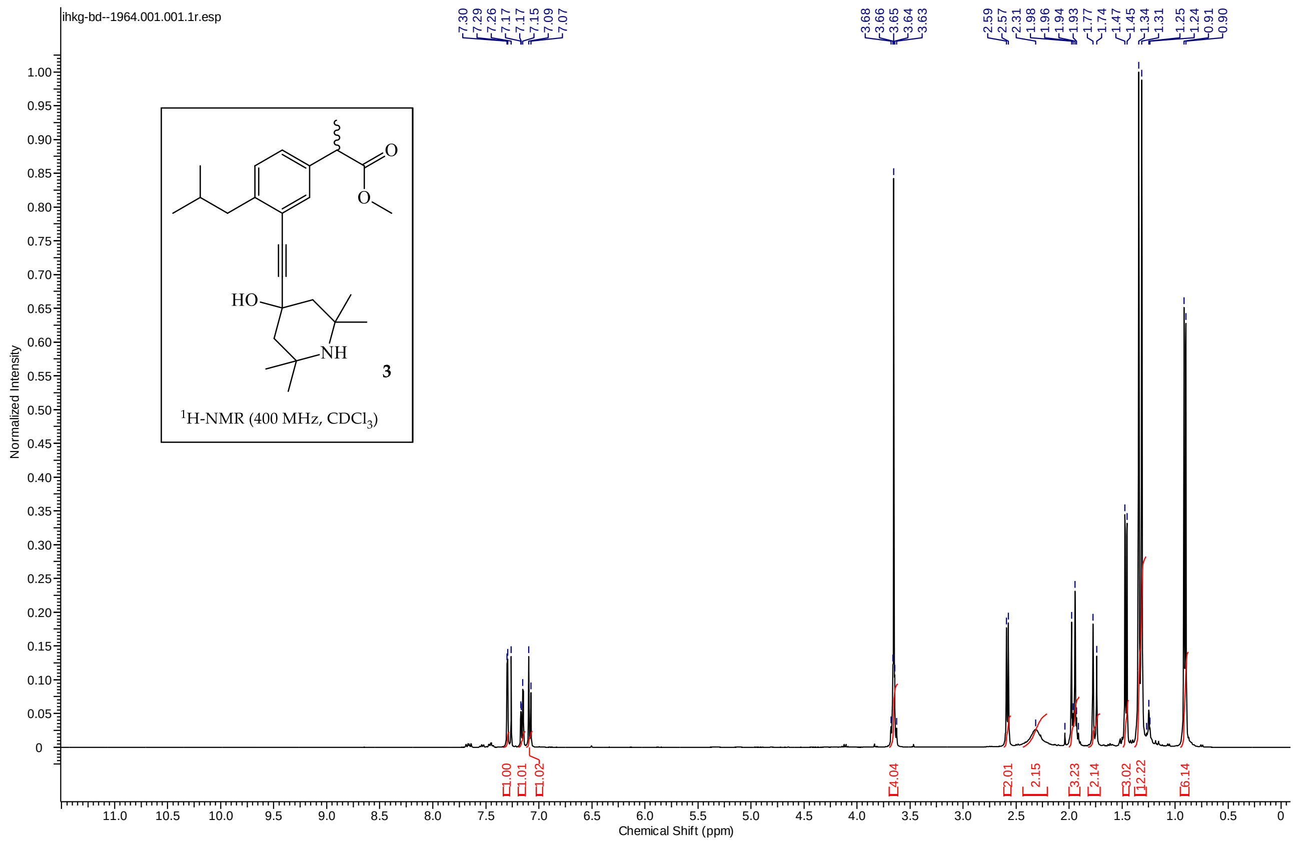

Figure S10. ^{1}H-NMR spectrum of compound **3**.

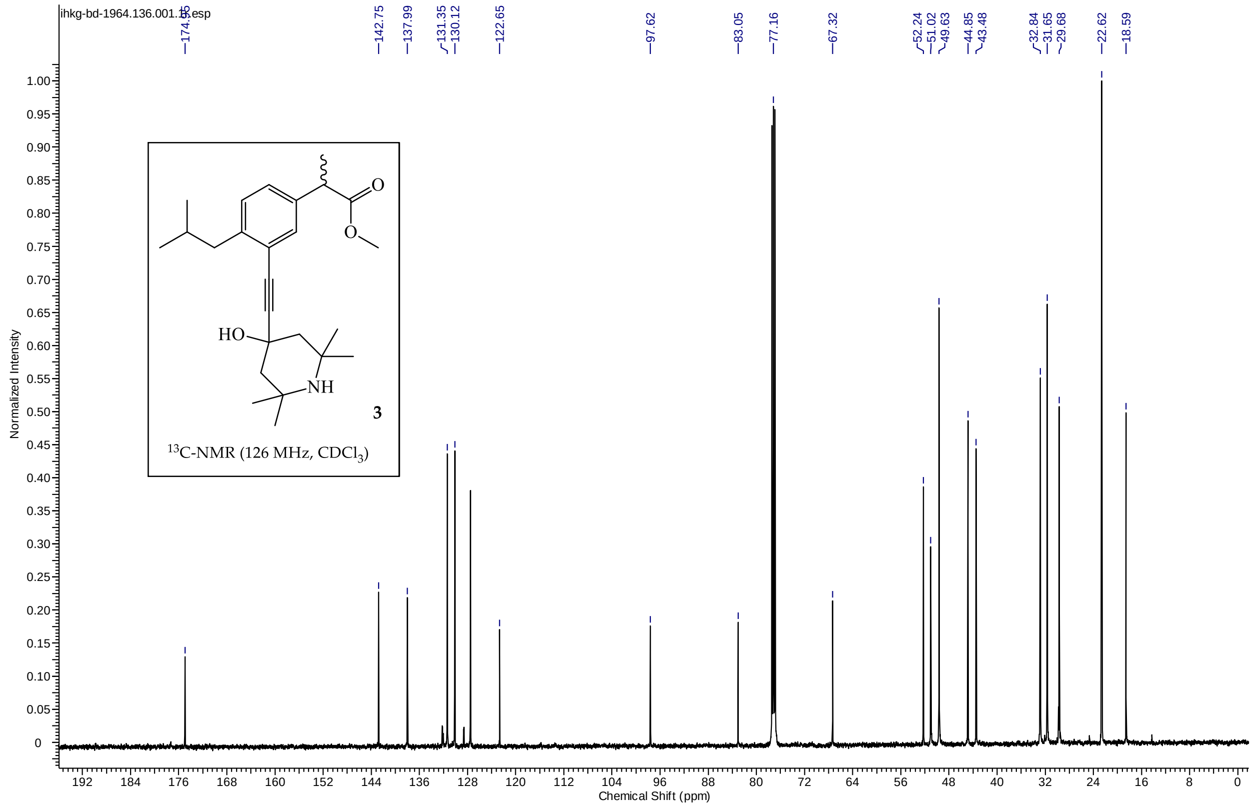

Figure S11. ^{13}C-NMR spectrum of compound **3**.

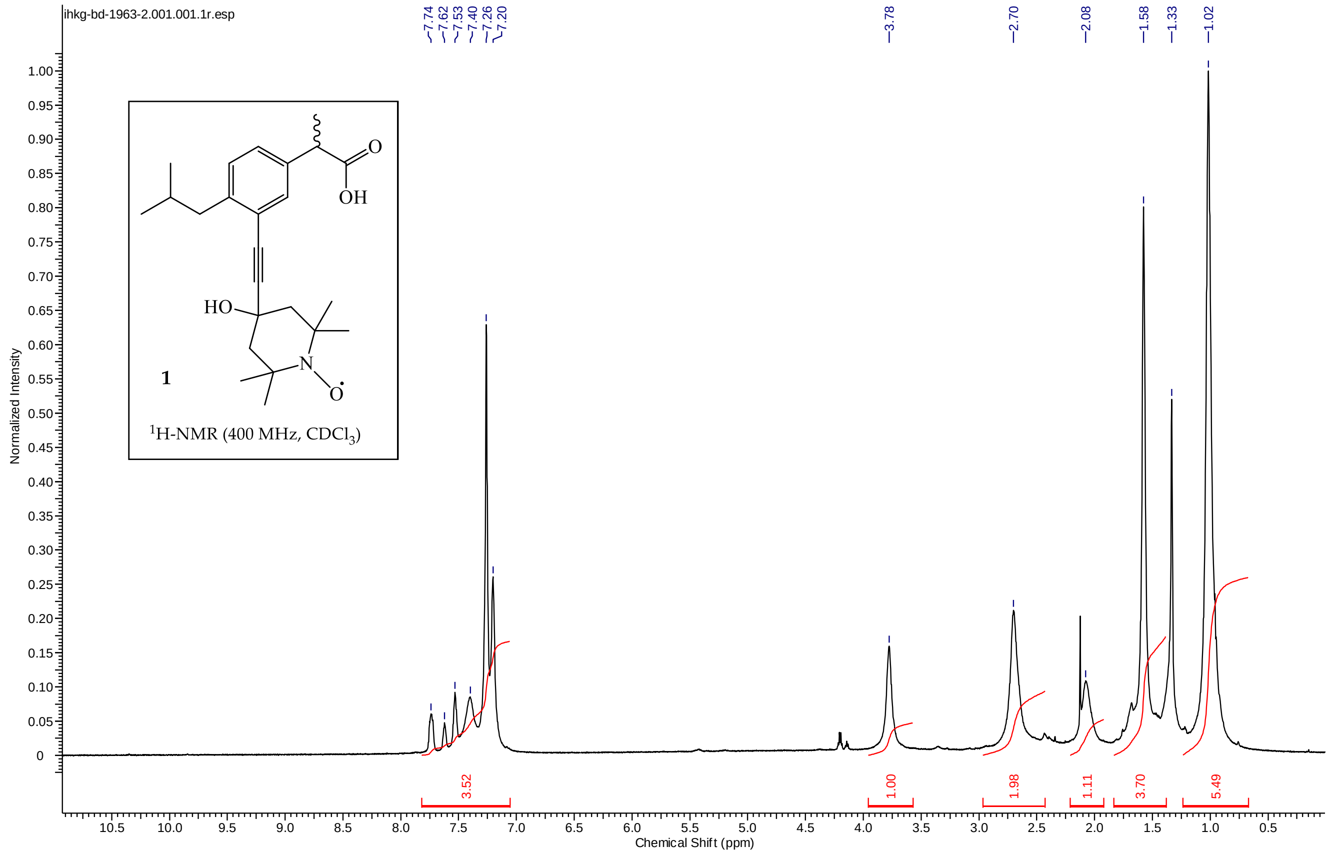

Figure S12. ¹H-NMR spectrum of compound **1**.

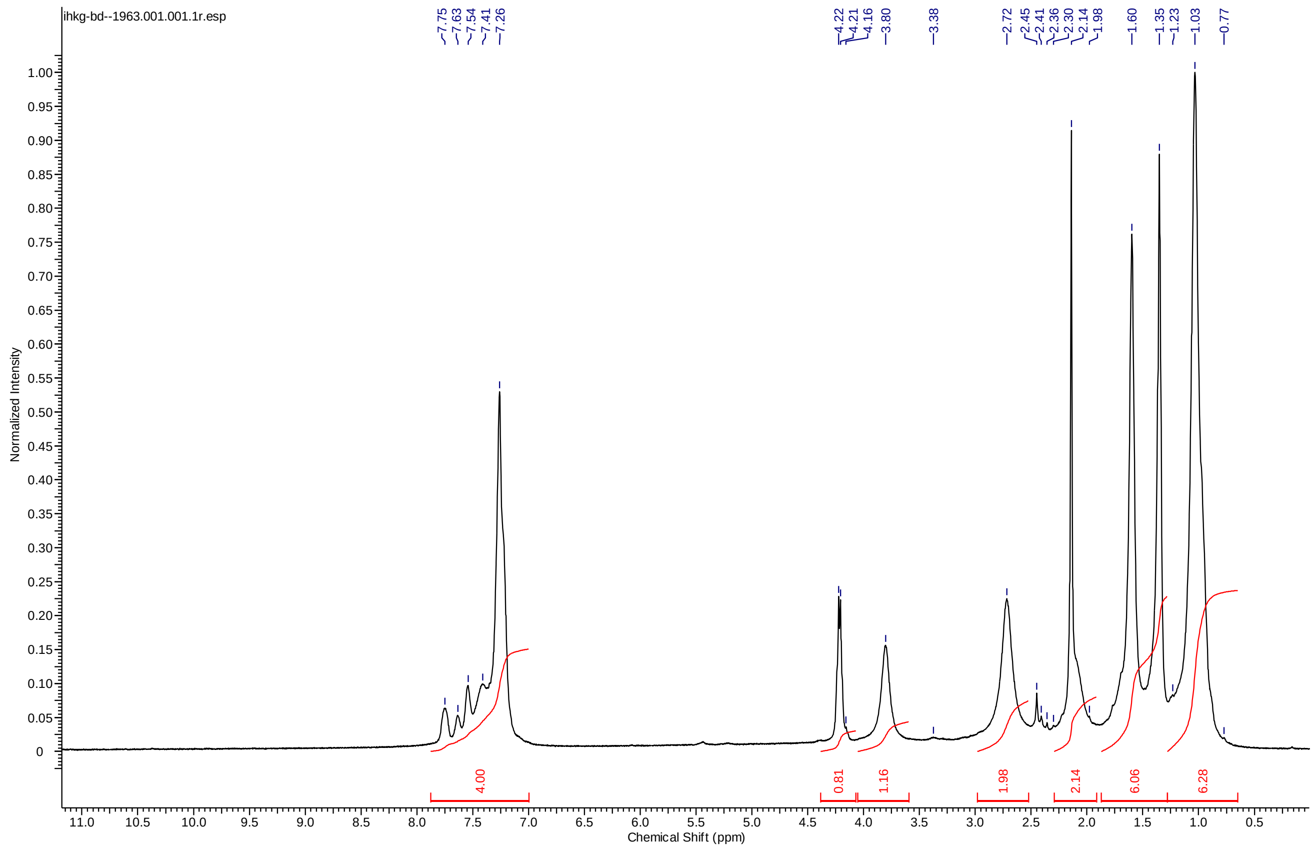

Figure S13. ¹H-NMR spectrum of mixture of compound **1** and ethyl acetate.

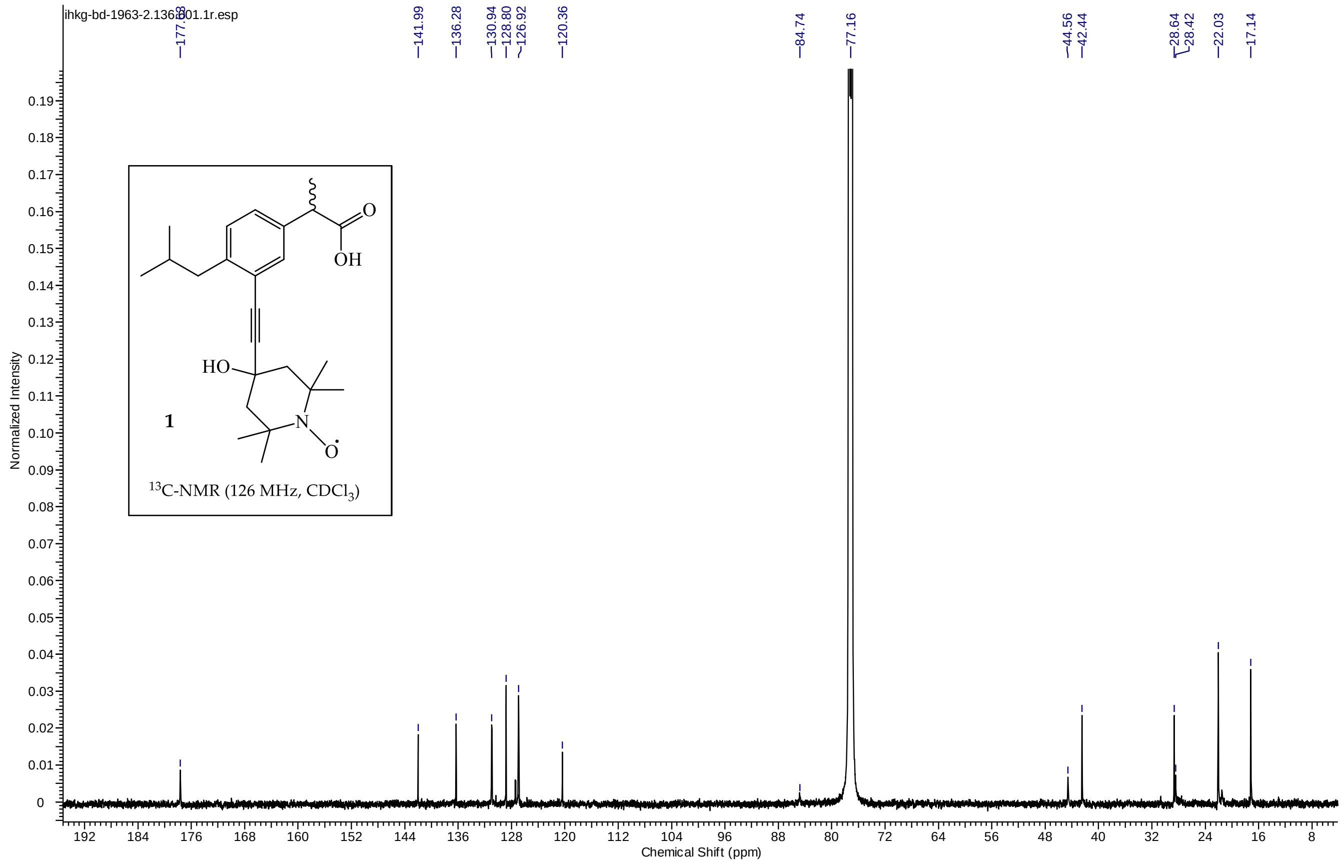

Figure S14. ^{13}C-NMR spectrum of compound **1**.

IR spectra

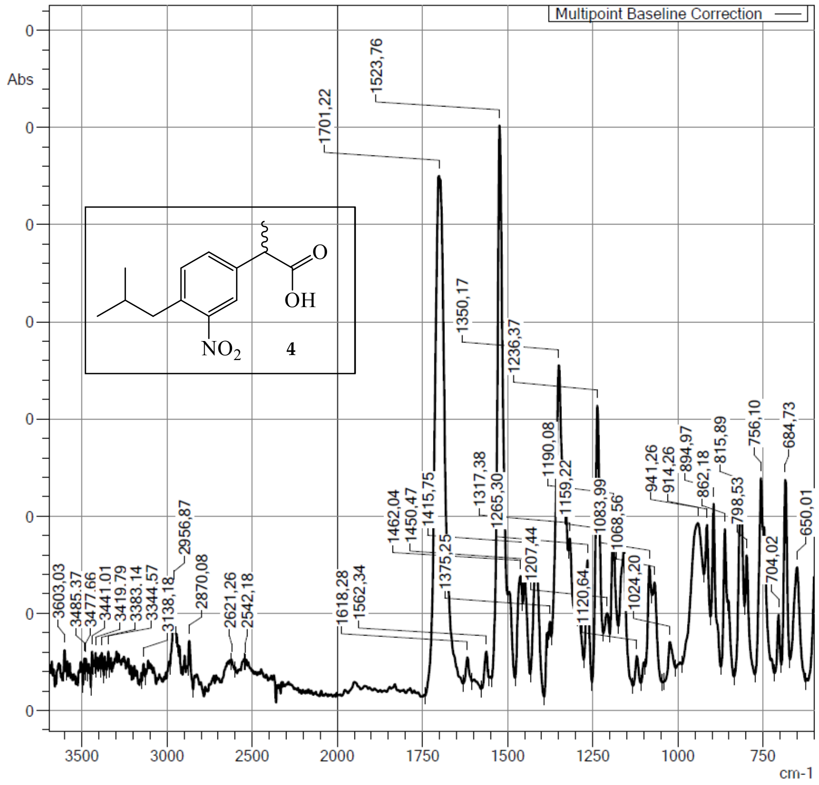

Figure S15. IR spectrum of compound **4.**

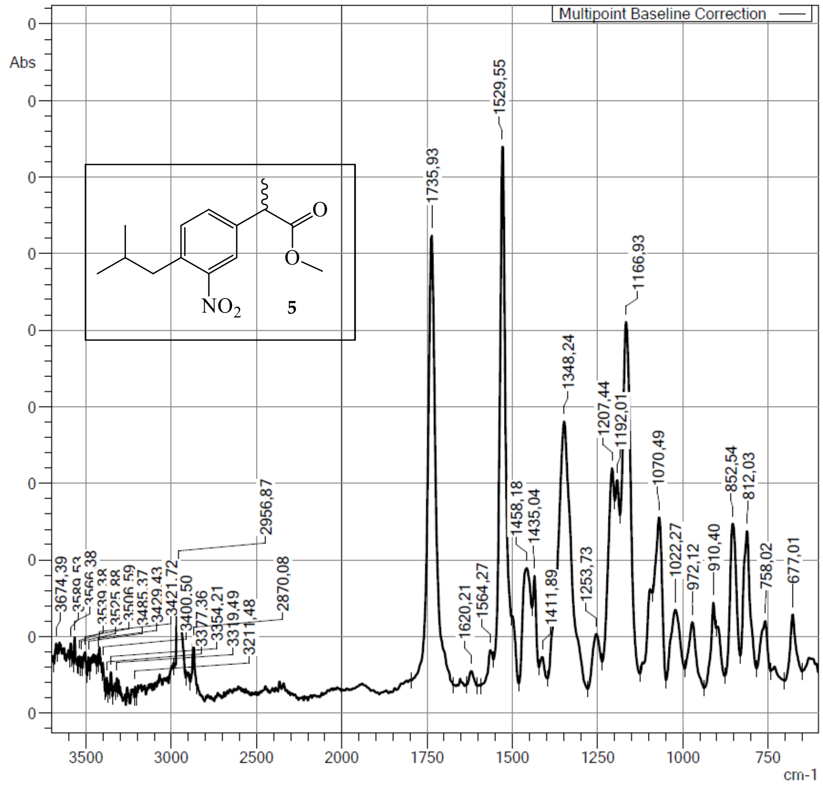

Figure S16. IR spectrum of compound **5.**

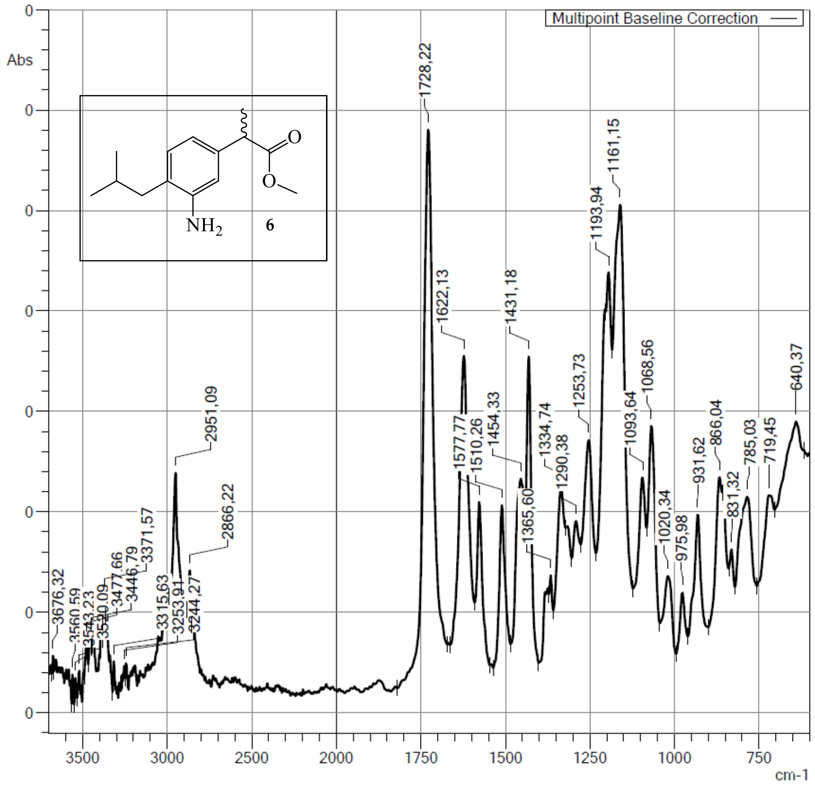

Figure S17. IR spectrum of compound **6.**

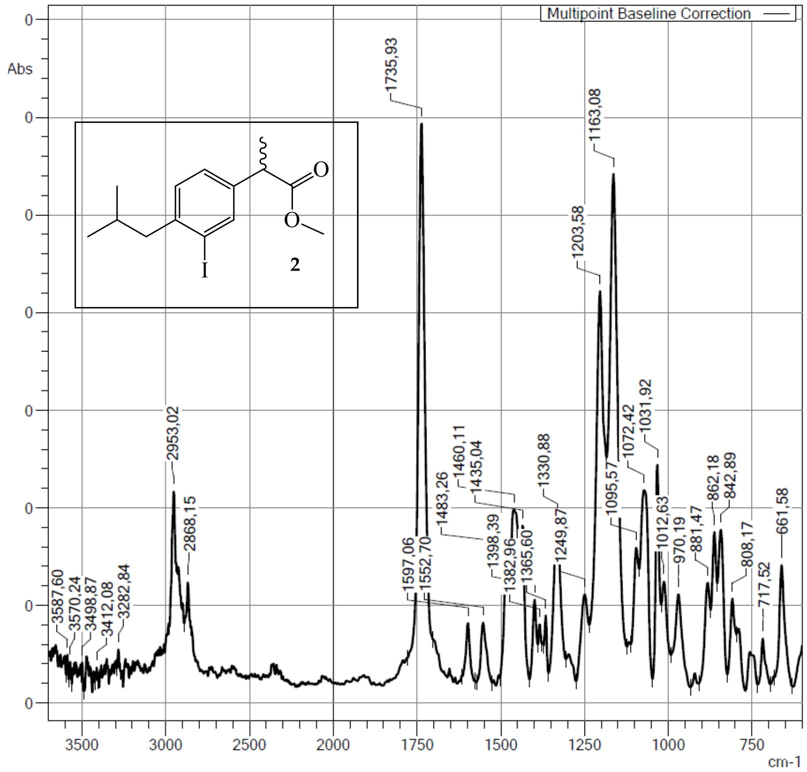

Figure S18. IR spectrum of compound **2.**

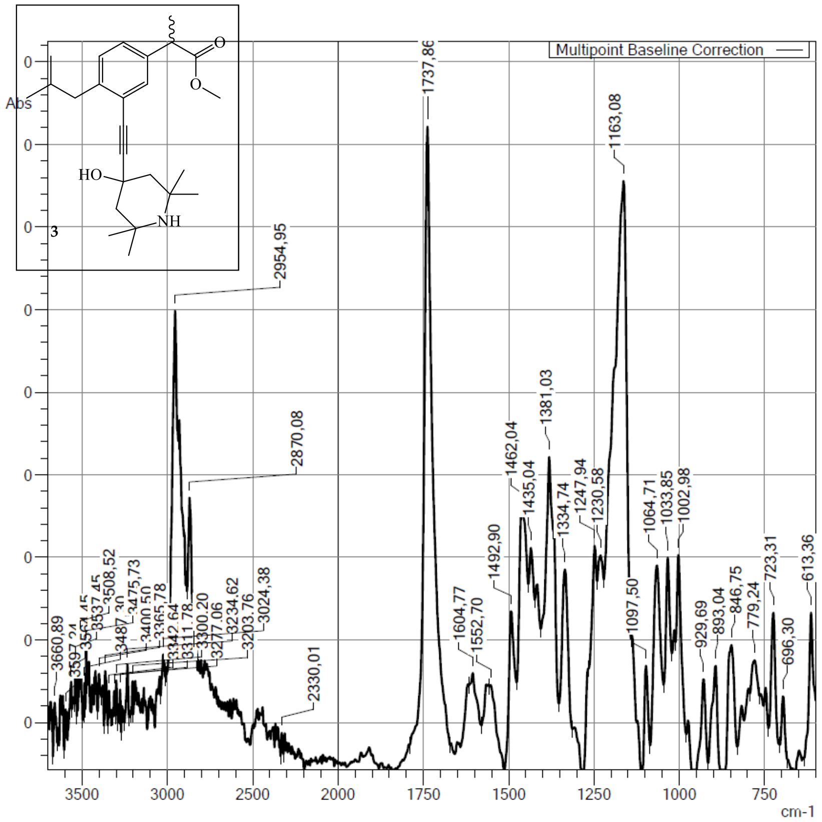

Figure S19. IR spectrum of compound **3.**

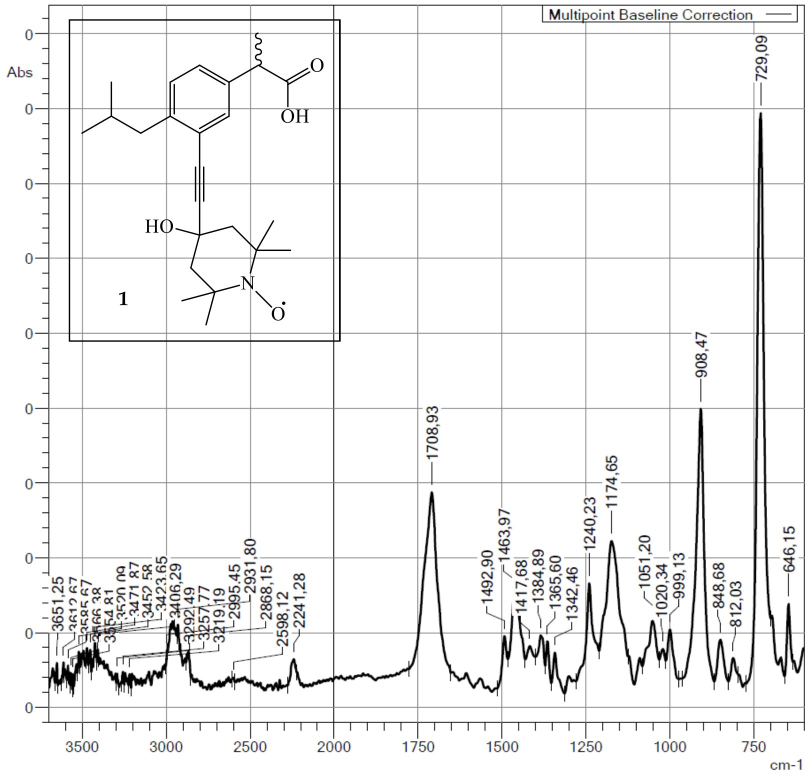

Figure S20. IR spectrum of compound **1.**

HRMS spectra

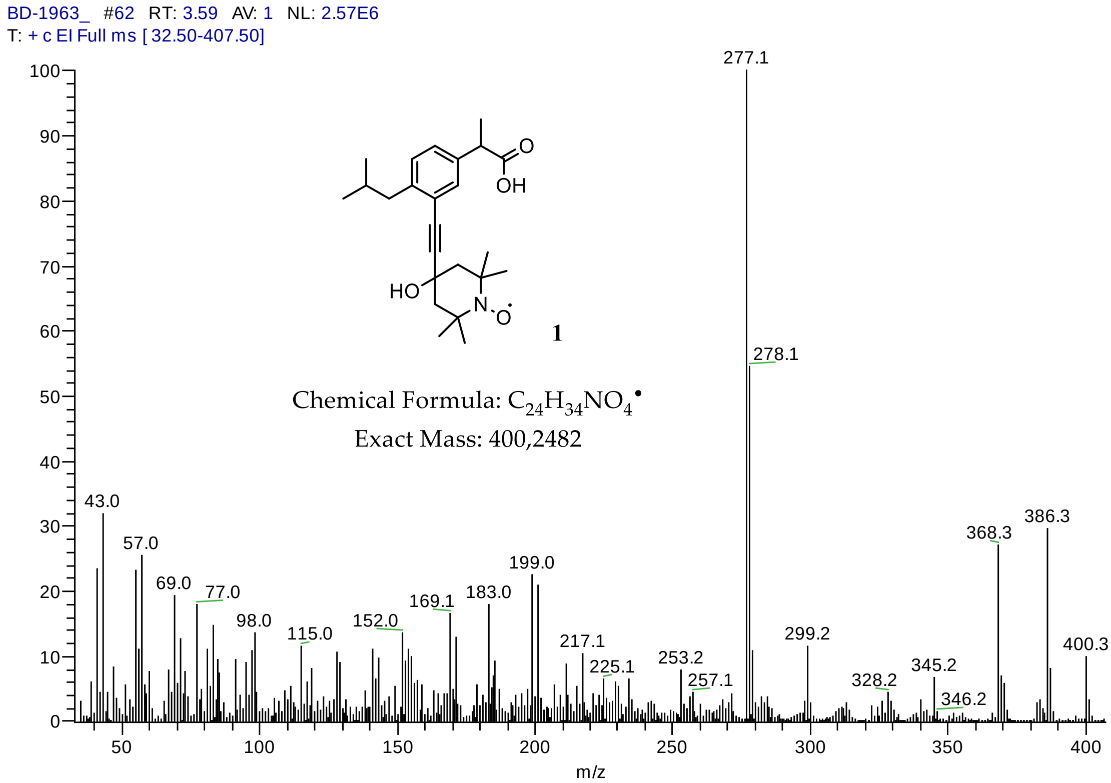

Figure S21. HRMS spectrum of compound **1.**

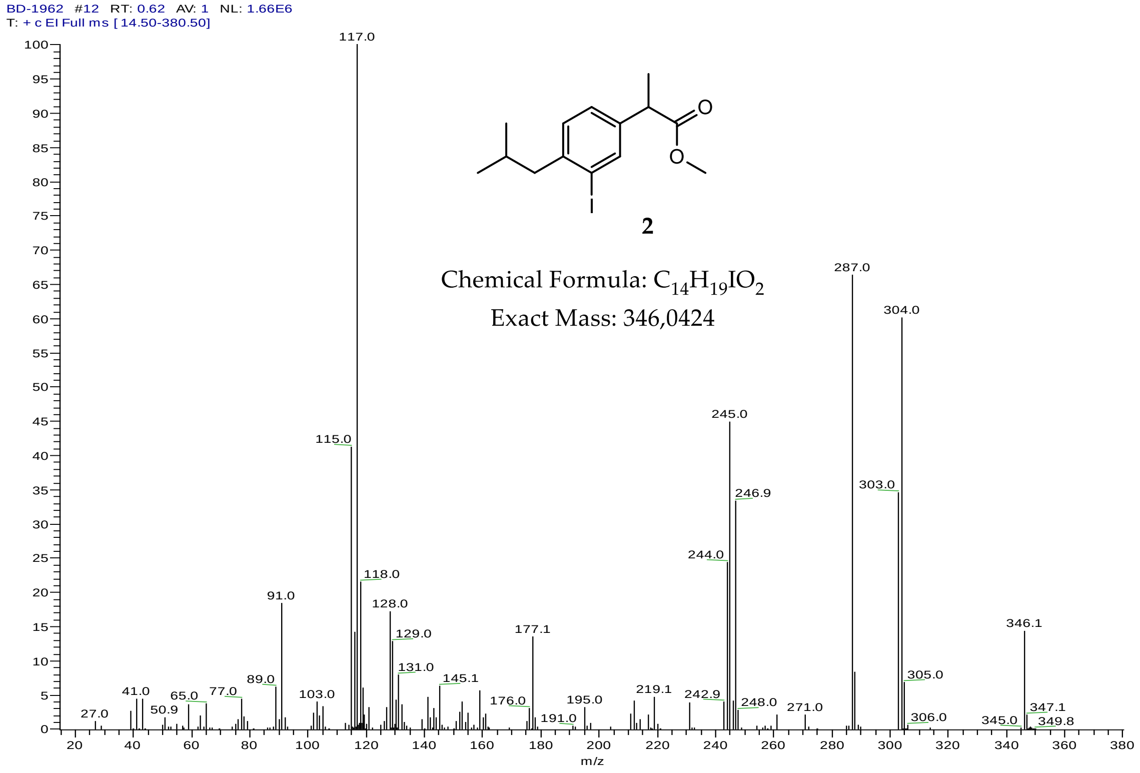

Figure S22. HRMS spectrum of compound **2.**

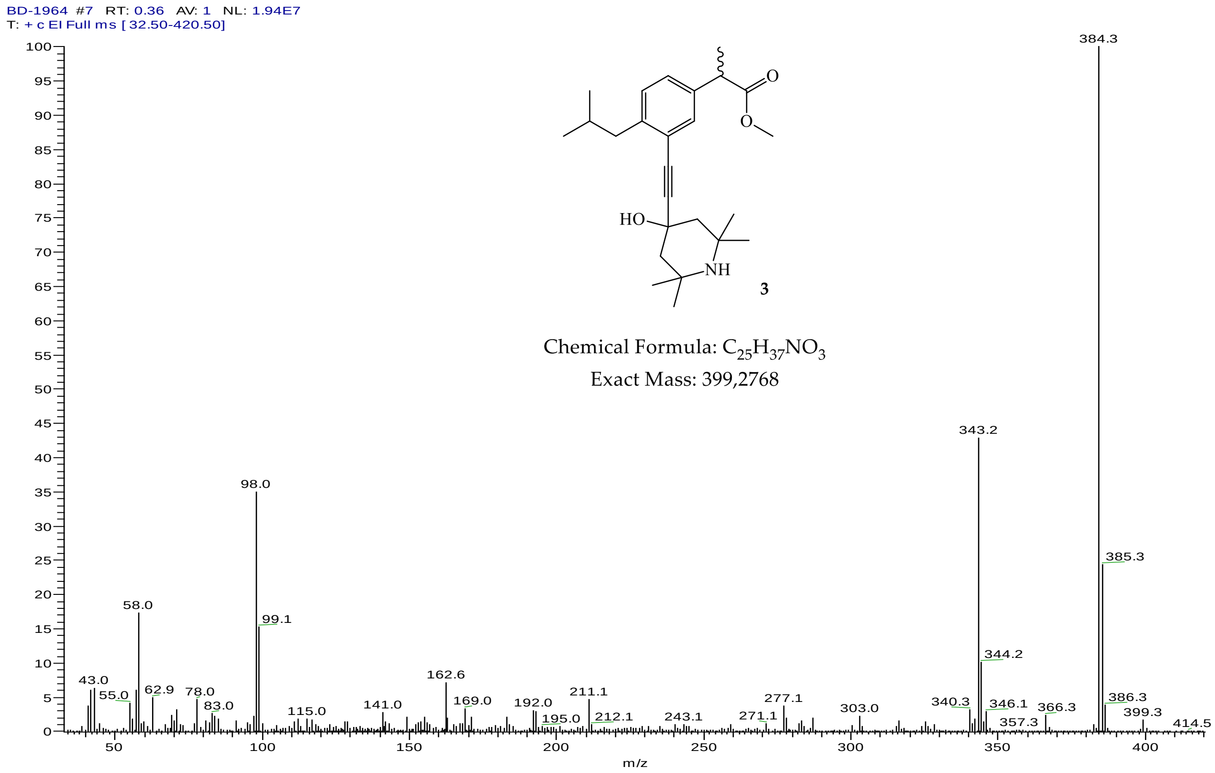

Figure S23. HRMS spectrum of compound **3.**

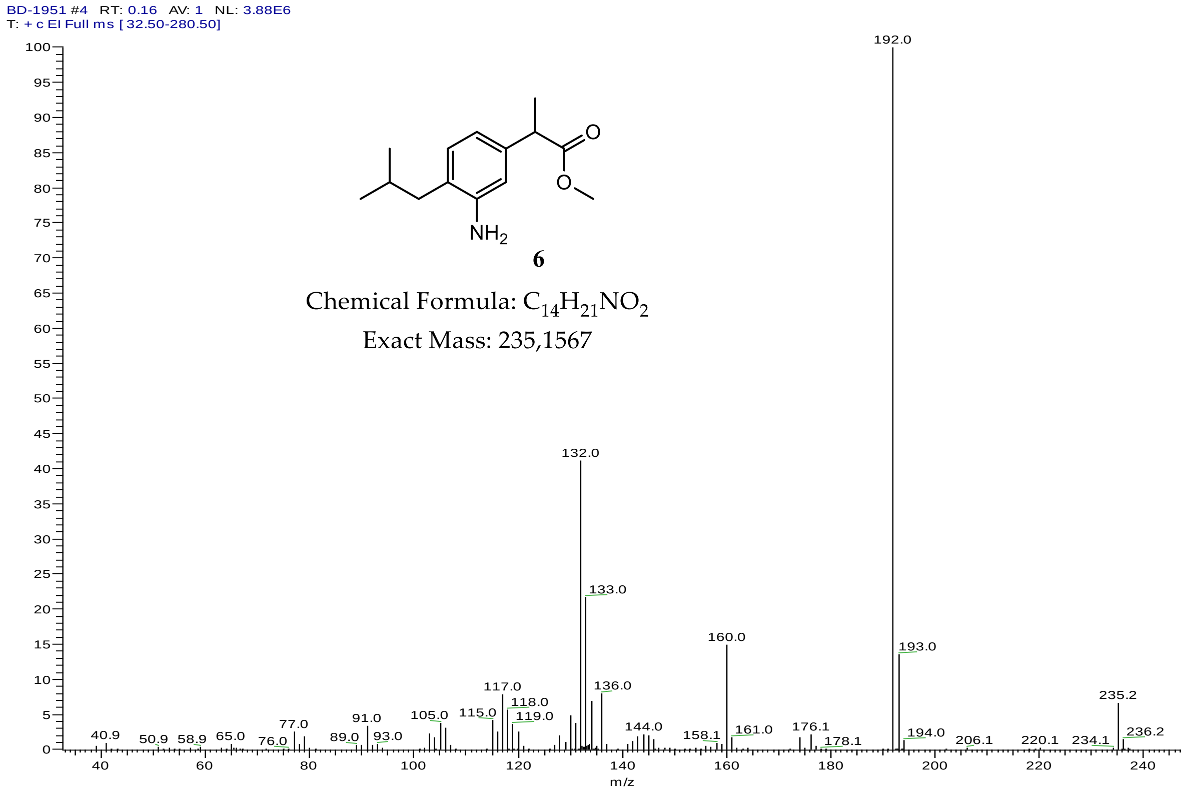

Figure S24. HRMS spectrum of compound **6.**
